# Improved Cook-stoves and Environmental and Health Outcomes: Lessons from Cross River State, Nigeria

**DOI:** 10.3390/ijerph16193520

**Published:** 2019-09-20

**Authors:** Robert Ugochukwu Onyeneke, Chinyere Augusta Nwajiuba, Jane Munonye, Uwazie Iyke Uwazie, Nkechinyere Uwajumogu, Christian Obioma Uwadoka, Jonathan Ogbeni Aligbe

**Affiliations:** 1 Department of Agriculture (Agricultural Economics and Extension Programme), Alex Ekwueme Federal University Ndufu-Alike, Ebonyi State 482131, Nigeria; munojane@gmail.com; 2 Department of Educational Foundations, Alex Ekwueme Federal University Ndufu-Alike, Ebonyi State 482131, Nigeria; caanwajiuba@gmail.com; 3 Department of Economics, Michael Okpara University of Agriculture Umudike, Abia State 440109, Nigeria; ui.uwazie@yahoo.com; 4 Department of Economics and Development Studies, Alex Ekwueme Federal University Ndufu-Alike, Ebonyi State 482131, Nigeria; ketchyus@yahoo.com; 5 Centre for Development Assistance Management, Partnership and Training, Alex Ekwueme Federal University Ndufu-Alike, Ebonyi State 482131, Nigeria; chrisuwadoka@hotmail.com; 6 Department of Planning and Policy Coordination, Federal Ministry of Agriculture and Rural Development, Benin City 300251, Nigeria; jonathan.aligbe@gmail.com

**Keywords:** fuelwood cook-stove, adoption, impact, health and environmental outcomes, inverse propensity score matching

## Abstract

This paper studies a topic in the triangle of environment, development and health—the effectiveness of the improved cooking solution. While a range of improved cook-stoves (ICS) is available in the market, since decades with a number of new entrants from recent years, adoption is still low in many developing regions, such as sub-Saharan Africa, also because stove performance is sometimes found to be deficient. However, in Nigeria, Africa’s most populous country, few improved cook-stove interventions are on-going. Incidentally, there is little evidence on the effect of improved cook-stoves on different components of health and environmental outcomes in rural Nigeria. This study, using cross-sectional data from Cross River State, the State with the largest forest area in the country, analyzed the impacts of locally designed improved cook-stoves on the environmental and health outcomes of rural women. A sample of four hundred (400) married women was drawn from eight rural communities with the highest concentration of improved cook-stove users. The woman in-charge of cooking in each household was the respondent. Also, in each household, the household head (if different from the primary cook) was interviewed. Using inverse propensity score weighting for data analysis, we found significant fuel and time savings from the adoption of the cook-stove. However, this study found no evidence of the reduction of indoor air pollution associated diseases given stove design and users’ behavior. This underscores the need to revisit the design of the stove and consider users’ cooking behavior in the design.

## 1. Introduction

There is ample evidence that about two billion persons in the world use biomass fuels in cooking and heating [1,2,3]. Sub-Saharan Africa constitutes a greater part of this population. For example, in Nigeria, Africa’s most populous country, about 72 per cent of the inhabitants use fuelwood for cooking [4,5]. The use of fuelwood for cooking causes significant negative impacts on the health and environmental outcomes of Africans. This is because the use of fuelwood in inefficient ways causes deforestation, stress resulting from increased time for cooking and fuelwood gathering, climate change and indoor air pollution leading to respiratory infections and diseases and eye diseases [6,7,8,9,10,11,12,13,14]. The World Health Organization (WHO) report on the Burden of Disease from Household Air Pollution for 2016 records 3.8 million deaths annually from cooking with biomass fuels [15]. An important strategy to reduce the prolonged time for cooking and gathering fuelwood, diseases and deaths associated with indoor air pollution, climate change and deforestation and improve productivity is an introduction and use of improved cook-stove (ICS) [12,16,17,18,19,20,21]. Use of improved cook-stove minimizes the cost of purchasing fuelwood, shortens the time required for cooking and fuelwood collection [14,22,23,24,25,26,27].

However, despite the multiple economic, social, environmental, and health benefits of improved cook-stoves (ICS) enumerated above, the adoption is still very low in Nigeria, particularly, and Africa generally [28]. Many researchers have cited socioeconomic, cultural and political barriers as the main causes of the low adoption rate of improved cook-stoves in Nigeria. There are some efforts in Nigeria by governmental and non-governmental organizations in disseminating different improved cook-stoves in Nigeria [29,30,31,32,33]. These efforts have not been sufficient because of their poor reach to rural women in communities where the use of firewood is high. This led to the promotion of locally designed improved cook-stoves in rural farming communities in Cross River State with one of the largest forest areas in Nigeria. In such communities, the locales design improved wood cook-stove using local materials, such as clay and water.

There is a growing literature on the impacts of improved cook-stove on different household health and environmental outcomes across the world (including on time spent on fuelwood collection, time spent on cooking, fuelwood use, as well as health impacts). These include Onyeneke et al. [14], Hanna et al. [34], Bensch et al. [35], Bensch and Peters [36], Bensch and Peters [37], Bensch and Peters [38], Hanna et al. [39], Burwen and Levine [40], Brooks et al. [41], Pattanayak et al. [42], Adrianzén [43], Beyene et al. [44], Ludwinski et al. [45], Rosa et al. [46], Smith-Sivertsen et al. [47], and Nepal et al. [48]. One big remaining question is why adoption rates remain low and the drivers of low adoption for these devices, despite considerable evidence on their efficacy [49] especially in Africa’s most populous country, Nigeria. Also, the literature on the effectiveness of improved cook-stoves in improving the health and environmental outcomes of rural households is mixed because of the variable performance recorded by different ICS [34,39,42,48,49,50,51,52,53]. This calls for further research on the effect of the use of improved cook-stoves in rural communities in Africa’s most populous country, where dependence on firewood for cooking is high. The study, therefore, analyzed the determinants of the use of improved cook-stoves by rural women and the effect of the use of improved cook-stoves on different components of environmental and health outcomes of rural women in Cross River State, Nigeria. The study is crucial for understanding the effects of the use of improved cook-stoves on the social, health and environmental spheres of rural communities in Nigeria. The lessons from this study will benefit policymakers in developing a pro-poor strategy for transitioning to clean cooking solutions in rural areas.

## 2. Methodology

### 2.1. Description of the Study Area

**Figure 1:**
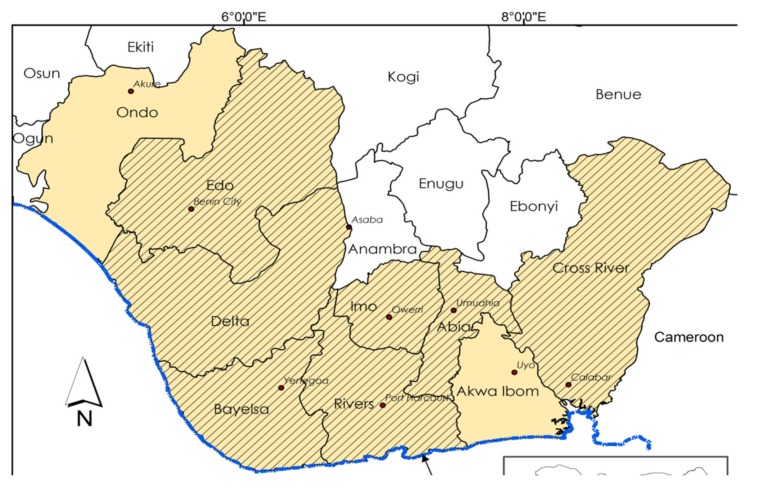
Map of the Niger Delta.

This study was conducted in Cross River State—an important State in Nigeria’s rainforest and coastal region. The last census held in Nigeria put the population of Cross River State at 4,219,244 persons [54]. About 73.3 per cent of households in the State depends on fuelwood as their primary cooking energy source [4]. Cross River and the other Niger Delta States record high indoor air pollution (IAP) level from inefficient, traditional stoves [55,56]. Figure 1 is the map of the Niger Delta area. The dependence on fuelwood for cooking is higher in poorer and rural areas of the State, and Cross River is one of the poor States in Nigeria, with a poverty rate of 60.4 per cent [4,57]. The report of the Nigerian Infrastructure Advisory Facility indicated that about 10,000 improved cook-stoves were in use in some rural communities in Cross River State [58].

### 2.2. Sampling Technique and Data Collection

Using a rapid survey of some stakeholders in the cook-stove sector, the authors identified the communities with a high concentration of wood cook-stove users. The design of the improved cook-stoves used in the communities was largely consistent across the study participants. The cook-stoves were made from 100% local materials - mud, clay and other locally sourced materials in the communities. The stoves have an opening where fuelwood is loaded before cooking.

The authors selected eight communities with the highest concentration of wood cook-stove users for further study. Cross-sectional survey was conducted by the authors in the eight communities. The authors first conducted a random sampling of women in the chosen communities and selected ten users of wood cook-stove without replacement in each community. After the selection of users, the authors selected forty non-users in each community. This implies that a sample of fifty married women was drawn randomly in each of the eight selected communities - ten women that used the stove (users) and forty that did not use wood stoves (non-users). The stoves had been already used in these families when the study started. This brought the sample size of this study to 400. Also, the use of improved cook-stove among rural women in some communities in Cross River State is low [5,29,58]. This was the reason why the authors chose the ratio of one is to four in sampling respondents for the study as being sufficient representation of the proportion of users and non-users, respectively in the communities. The woman in-charge of cooking in each household was the respondent. Also, in each household, the household head (if different from the primary cook) was interviewed. Cross-sectional data on stove use, time spent on cooking and fuelwood collection, fuelwood use and health outcomes were collected. Also, data of the socioeconomic characteristics of the households and women were collected.

### 2.3. Impact Pathway

Impact estimation is always confronted by two serious biases—overt and hidden biases [59,60,61]. The literature recognizes two biases in impact estimation known as overt bias (selection on observables) and hidden bias (selection on unobservables) [59,60,61]. This study adopted the inverse propensity score weighting (IPSW) technique to deal with these problems. The IPSW estimates are the average treatment effect (ATE), the average treatment effect on treated (ATT) and average treatment effect on non-treated (ATT0) [62] (Rosenbaum and Rubin, 1983). Their formulae, as stated in the works of Lee [59], Imbens [63] and Diagne and Demont [64], are stated, thus:

(1) $$\mathrm{ATE}=\frac{1}{n}\sum_{i=1}^{n}\frac{(\left(m_{i}-p\left(x_{i}\right))y_{i}\right)}{p(x_{i})\;\left(1-p(x_{i}\right))},$$

(2) $$\mathrm{ATT}=\frac{1}{n_{1}}\sum_{i=1}^{n}\frac{(\left(m_{i}-p\left(x_{i}\right))y_{i}\right)}{\;\left(1-p(x_{i}\right))},$$

(3) $$\mathrm{ATT}0=\frac{1}{n_{1}-1}\sum_{i=1}^{n}\frac{(\left(m_{i}-p\left(x_{i}\right))y_{i}\right)}{p(x_{i})},$$

where,

*n* is the sample size
*n*_1_ = $\sum_{i=1}^{n}m_{i}$ is the users
*p*(***x***_i_) is the propensity score estimate evaluated at *x.*
$m_{i}$ is the treatment variable
$y_{i}$ = outcome variables
ATE = is the average effect of the use of the cook-stoves in the population.
ATT = is the average effect of the use of the cook-stoves on the subpopulation of users.
ATT0 = is the average effect of the use of the cook-stoves on the subpopulation of non-users. This is important and can be used to gauge the spillover effect of the program. That is to track the potential non-users that adopted or might adopt the cook-stoves in the future.

In estimating the propensity score, the binary probit model was adopted.

The probit model is written as:

(4) $$P(x_{i})=\mathrm{Pr}(m=1|x_{i})=f(x_{i}{}^{\prime }\beta )=\int_{-\infty }^{x^{\prime }\beta }ɸ\left(z\right)dz.$$

ɸ(*z*) = $\frac{1}{\sqrt{2\pi }}e^{-\frac{z^{2}}{2}}$ is the density function of the standard normal distribution, m is a binary endogenous variable with value 1 if the woman is a user of any locally designed improved cook-stove and 0 otherwise, *β* is the vector of parameter estimates, and *f* is a cumulative density function. *x_i_* is the vector of independent variables (time-invariant characteristics), which include:

X_1_ = Educational level (number of years spent in school)
X_2_ = Spouse alive (dummy variable, yes = 1, no = 0)
X_3_ = Age (years)
X_4_ = Household size (number of persons)
X_5_ = Income (Naira)
X_6_ = Access to credit (amount of credit in Naira borrowed by the household)
X_7_ = Forest area (total area of forest in hectares owned/controlled by the woman)
X_8_ = Accessible road near the household (accessible road = 1; no accessible road = 0)
X_9_ = Membership of women association (dummy variable, member = 1, non-member = 0)
X_10_ = Major occupation (dummy variable; agriculture = 1; otherwise = 0)
X_11_ = Preference for the taste of food prepared with traditional cook-stoves over-improved cook-stoves (dummy variable, yes = 1, no = 0).

## 3. Results and Discussion

### 3.1. Women Socioeconomic and Kitchen Characteristics

Table 1 contains a summary of the socioeconomic characteristics of the women studied. The users were the women using any locally designed improved cook-stove, while the non-users did not adopt/use the stove. As stated in earlier, bias is one of the major problems confronting impact assessment of interventions. The authors further controlled this problem by presenting the summary statistics of the women interviewed and disaggregated the results according to users and non-users to ascertain whether they are comparable. The average age of the women in the users’ group was 40.33 years, while that of the non-users was 41.80 years. The users spent an average of 6.84 years in school, while the non-users spent an average of 5.74 years. Ninety percent of the users had their spouses alive, and this was significantly higher than the share of spouses alive (73 per cent) among the non-users (*p* < 0.01). The average household size of the users was approximately ten persons (9.51 persons), while the average household size of the non-users was approximately eight persons (7.55 persons). There is a statistically significant difference between the household sizes of the two groups (*p* > 0.01). The users of improved cook-stoves had an average annual income of N317,500.00, which was significantly higher than the average annual income of the non-users (N188,013.50). The women were the primary cooks (98.00% for the users and 100.00% for the non-users). Twenty per cent (20.00%) of the women in the users’ group had their kitchen enclosed indoors in the living area with partition and windows, while it was 22.00% for the non-users. Furthermore, the majority (78.00% of the users and 76.00% of the non-users) of the women had a separate indoor kitchen outside the living area with windows attached. These characteristics, which represent kitchen ventilation of the women and the insignificant results of the respective t-tests, showed that there were no differences in the type of kitchen and corresponding ventilation enjoyed by the users and non-users. The characteristics of the two groups showed that most of the variables presented were statistically indistinguishable from their respective t-ratios, indicating that the two groups were similar and comparable. This result is very consistent with that of Onyeneke et al. [14] and Sagbo and Kusunose [53].

### 3.2. Drivers of the Use of Improved Cook-Stoves

The binary probit regression result is presented in Table 2. The model is fit, and the demographic and household characteristics of the women jointly explained use of the improved cook-stove decisions of the women. This is as a result of the significance of the likelihood ratio Chi Square (Chi^2^) of 223.81 (significant at 1% level). Nine out of the eleven independent variables were statistically significant. These include a woman having spouse alive, age, household size, income, credit, forest area controlled, presence of accessible road, member of women organizations, and preference for the taste of the food prepared with traditional cook-stoves over-improved cook-stoves.

Women whose spouses were alive used the improved cook-stoves more than the women whose spouses were dead. This could be due to the fact that women whose husbands are alive stand a better chance of receiving support and advice related to technology adoption/use than their counterparts whose husbands are dead. Age and household size significantly emerged as significant predictors of the use of improved cook-stove. While age yielded a significant negative impact on the use of improved cook-stove, household size affected its use positively. This implies that younger women used improved cook-stove more than aged women, while women with a greater number of persons in their households to feed used improved cook-stoves more readily than their counterparts with a smaller number of persons in their households to feed. These results are in agreement with the findings of Alem et al. [65], Narasimha and Reddy [66], Ouedraogo [67] who found the household size to increase uptake of improved cook-stove. Similarly, Onyeneke et al. [68] and Adrianzén [69] found a significant negative impact on the adoption of improved cook-stove in Africa and Latin America, respectively.

Income and access to credit significantly increased the uptake of locally designed improved cook-stoves in Cross River State. Though the locally designed cook-stoves are affordable, it requires little cost to install a unit, and credit availability enhanced the use of the technology. The finding substantiates the research results of Onyeneke et al. [14], Gebreegzabiher et al. [70] and Beyene and Koch [71] who found income and credit to increase the adoption of improved cook-stoves in Africa. This is also similar to the finding of Mamuye [72] in Ethiopia who recorded that finance and stove price determine adoption.

Forest area controlled or managed by women yielded a significant and negative effect on the use of improved cook-stoves in the area. This implies that women controlling fewer forest areas used improved cook-stoves more than their counterparts controlling larger forest areas. Women that had/controlled larger forest area had a higher quantity of fuelwood at their disposal and may have considered fuelwood savings less important and less attractive. Also, the opportunity cost of labour in rural areas is usually low, and even when it is said that the improved cook-stoves are efficient by reducing cooking time and fuel collection time, the women do not appreciate this because they may not invest the time saved in productive/income-generating activities.

Accessible road(s) close to a woman’s house significantly increased uptake of improved cook-stoves in Cross River State. One can infer that the women users lived close to accessible roads and were at an advantaged position to assess the market and easy transportation means to access improved cook-stoves. Membership of women associations had a positive and significant impact on the use of improved cook-stoves. This is expected because many local entrepreneurs often work with cooperative societies, community-based associations or faith-based organizations to reach members of such associations/organizations. Members of such organizations often get information about innovations/technologies/inventions from their organizations. This re-echoes the role of social organizations/institutions in technology promotion, dissemination, adoption and use. Vulturius and Wanjiru [73] documented the role of social relations in the adoption of improved cook-stoves. Link et al. [74] found that household’s access to community-based organizations affects the adoption of clean fuels and cook-stoves.

Preference for the taste of food prepared with traditional cook-stoves over-improved cook-stoves was found to slow use of improved cook-stoves in the area. Food tastes and cooking practices have been documented to affect the uptake of improved cook-stoves. In Mexico for instance, rural dwellers preferred cooking “tortillas” with traditional biomass stoves over-improved cook-stove because it affects the taste [75].

### 3.3. Inverse Propensity Score Weighting Estimates

In the course of data collection, stove stacking (combining improved cook-stove and traditional open fire in cooking) was noticed in few user households. Such stacking mainly happened when there was a large volume of cooking, especially for social events. Social events do not happen frequently/regularly. Furthermore, even when we included non-exclusive/exclusive use of the improved cook-stove as a proxy for stacking in the inverse propensity score weighting analysis done, the exclusive/non-exclusive use variable and age/duration (time since stove installation) of the improved cook-stoves variable were dropped by the model suggesting possible multicolinearity problem with some other variables. Hence, the authors did not consider the variables in the final analysis.

The results of the estimation of the impact of the use of improved cook-stoves on certain women’s welfare outcomes (such as average daily time spent on cooking, gathering of fuelwood, quantity of fuelwood used in cooking and incidence of ailments associated with indoor air pollution as a result of cooking with fuelwood) using inverse propensity score weighting is presented in Table 3. The table shows that as a result of using improved cook-stove, the users saved an average of 1.44 h per day in cooking and 0.75 h per day in fuel collection. The daily fuelwood savings for these women was 1.29kg. The cook-stoves also demonstrated potential fuelwood and time savings on the entire population (ATE) and the subpopulation of women who are yet to use the stove. The daily time of fuelwood collection time was saved because of less fuelwood required for cooking as compared to using a traditional device. The improved cook-stoves were efficient in reducing forest degradation through harvesting of fuelwood for cooking, saves time, which rural women can have for rest or re-invest into various income-generating activities. The literature is replete on the fuelwood and time savings attributes of improved cook-stoves. For example, DeWan et al. [76] averred that adoption of improved cook-stove led to a significant reduction in fuelwood consumption (40.1%), fuelwood collection (38.2%), and tree felling (23.7%) in China. Similarly, Bielecki and Wingenbach [77] and García-Frapolli et al. [16] documented that improved cook-stoves saved fuelwood and decreased fuelwood collection time. Bwenge [78] in Tanzania found that improved cook-stoves saved fuelwood consumption and reduced time spent on fuelwood collection. Okuthe [79], Bensch and Peters [36], Bensch and Peters [37] and Honkalaskar et al. [80] studies depicted that adoption of improved cook-stoves led to a significant reduction in time for cooking meals in different parts sub-Saharan Africa.

The authors evaluated the impact of the cook-stove on the incidence of self-reported ailments (cough and sore eyes) associated with indoor smoke exposure. Reductions in self-reported cough and sore eyes in the entire population and subpopulation of users were recorded. Incidentally, the reductions were statistically insignificant. The reason for the insignificant impact of the cook-stoves on self-reported ailments associated with smoke exposure from the cooking area is not far-fetched. It could be possible that the locally designed improved cook-stoves did not substantially reduce self-exposed carbon monoxide and particulate matter of the cooks in the cooking area mainly as a result of the material used in making the cook-stoves (mainly clay), as well as the design. It is, therefore, logical to state that this could have led to the simultaneous/sequential insignificant impacts on self-reported cough and eye sore recorded. This is our assumption, and further research in this area is needed to elucidate why the stove did not yield significant reductions in self-reported ailments associated with kitchen smoke exposure. Our result is similar to the findings of Romieu et al. [81], Smith-Sivertsen et al. [47] and Hanna et al. [34], who found lack of health impacts from the adoption of improved cook-stoves, which derive mainly from limited and improper use of the stoves.

## 4. Limitations

An important limitation of this study needs to be mentioned. The measurements of emissions of air pollutants from traditional and improved wood stoves, indoor tobacco smoking, second-hand smoke exposure and spirometry measurements were not possible because of huge costs involved in such tests/measurements, and some of the endpoints of the tests may take a long time to be completed. The authors resorted to self-reported health conditions, which several studies across the world have used. Further research can be conducted in this area to ascertain the extent to which measured indoor air pollution, second-hand smoke exposure and spirometry tests can affect the results of the use of improved cook-stoves on health and environmental outcomes in the area. Mindful of this limitation, the study generated important conclusions.

## 5. Conclusions

The paper evaluated the effect of the use of locally made improved cook-stoves on rural women’s welfare in Cross River State, Nigeria. The outcomes include fuelwood use, time spent on cooking and fuelwood collection and incidence of ailments associated with smoke inhalation. Using inverse propensity score weighting technique, we found that use of improved cook-stoves led to a considerable and substantial reduction in fuelwood consumption, time spent on cooking and fuelwood collection. These results echo previous studies and so suggest that additional emphasis on the use of this particular apparatus can bring tangible benefits, especially in reducing deforestation and increasing women’s time use and productivity.

Strikingly, we found no significant evidence that use of the stoves reduced ailments/diseases associated with indoor air pollution from cooking with biomass. The probable reason for this unexpected result is attributed to the stove usage by the cooks and the design of the stove. The paper concludes that the use of improved cook-stoves in real-world settings is significantly less positive than expected. Mindful of this finding, our results suggest that simply designing/promoting improved cook-stoves may not always improve health and indoor air pollution, outcomes especially when the stove is designed without considering the users’ needs. Instead, policymakers and improved cook-stove designers/fabricators need to ensure that efforts to improve stove design and sell/disseminate the stoves should first consider the needs of the users and then be complemented by efforts to monitor compliance by users.

## Figures and Tables

**Table 1:** Women socioeconomic and kitchen characteristics.

Characteristic	Users (*N* = 80)	Non-Users (*N* = 320)	t-Ratio
	Average	Average	
Age (years)	40.33	41.80	−1.37 ^ns^
Educational level (years spent in school)	6.84	5.74	1.57 ^ns^
Spouse alive (share of husbands alive)	0.90	0.73	3.22 ***
Women having kitchen enclosed indoors in the living area with a partition (share)	0.20	0.22	0.34 ^ns^
Household size (persons)	9.51	7.55	4.80 ***
Annual income (Naira)	317,500.00	188,013.50	5.85 ***
Primary cooks (women share)	0.98	1.00	0.09 ^ns^
Women having a separate indoor kitchen outside the living area (share)	0.78	0.76	0.08 ^ns^
Women having open-air kitchen outside the living area (share)	0.02	0.02	0.01 ^ns^

Note: ^ns^ Not significant; *** Significant at 1 level.

**Table 2:** Probit estimates of factors affecting use of improved cook-stoves.

Variable	Coefficient	z-Value	Marginal Effect	z-Value
Education	−0.005	−0.26	−0.0008	−0.26
Spouse alive	0.704	2.60 ***	0.099	3.20 ***
Age	−0.038	−2.83 ***	−0.0068	−2.89 ***
Household size	0.121	3.99 ***	0.021	3.82 ***
Income	2.08 × 10^−6^	4.01 ***	3.70 × 10^−7^	3.74 ***
Access to credit	0.00002	5.86 ***	4.04 × 10^−6^	4.99 ***
Forest area	−0.973	−2.32 **	−0.173	−2.23 **
Accessible road near the household	0.657	3.12 ***	0.113	3.19 ***
Membership of women association	1.138	5.24 ***	0.258	4.55 ***
Major occupation	−0.205	−0.99	−0.036	−1.00
Preference for the taste of food prepared with traditional cook-stoves over ICS	−0.418	−1.90 *	−0.070	−2.01 ***
Constant	−2.277	−3.85 ***		
LR chi^2^(11)	191.12 ***			
Number of observations	400			
Prob > chi^2^	0.0000			

Note: *** Significant at 1% level; ** Significant at 5% level; * Significant at 10% level.

**Table 3:** Inverse propensity score weighting (IPSW) estimates.

Outcome	Unit	ATE	IPSW ATT	ATT0
Average daily cooking time	Hours	−1.45 (−3.71) ***	−1.03 (−2.30) **	6.381 (20.85) ***
Average daily fuelwood collection time	Hours	-0.75 (−11.23) ***	-0.73 (−9.47) ***	2.55 (41.04) ***
Average daily fuelwood consumption	kg/day	−1.28 (−3.87) ***	−1.638 (−22.13) ***	1.59 (4.80) ***
Average yearly number of cases of eye discomfort	Number	−0.35 (−0.27)	−0.40 (−0.73)	7.06 (5.55) ***
Average yearly number of cases of cough and catarrh	Number	−1.43 (−0.99)	−0.35 (−0.78)	9.19 (6.39)

Note: *** Significant at 1% level; ** Significant at 5% level; * Significant at 10% level. Values in parenthesis are z-values.
